# Robot-Assisted Surgery Performed Using a Fluorescent Ureteral Catheter for Locally Advanced Sigmoid Colon Cancer: A Case Report

**DOI:** 10.7759/cureus.108792

**Published:** 2026-05-13

**Authors:** Taiki Masuda, Shunsuke Miura, Sodai Arai, Misuzu Yamato, Mikito Inokuchi

**Affiliations:** 1 Department of Surgery, Japanese Red Cross Musashino Hospital, Tokyo, JPN

**Keywords:** colorectal cancer, fluorescent ureteral catheters, locally advanced colorectal cancer, robotic surgery, ureter injury

## Abstract

In recent years, robotic surgery for colorectal cancer has increased and is now being performed even for locally advanced colorectal cancers. However, due to adhesions and tissue fibrosis caused by infiltration and inflammation, the risk of misidentification during dissection is high. In robotic surgery, which lacks tactile feedback, particular caution is required to avoid injuries such as ureteral damage. Navigation surgery using fluorescent ureteral catheters that emit fluorescence upon near-infrared light irradiation has attracted attention. In this report, we describe a case where a safe robotic sigmoidectomy was successfully performed using fluorescent ureteral catheters. A 65-year-old man visited our hospital with a chief complaint of abdominal pain and was diagnosed with sigmoid colon cancer infiltrating the pelvic wall (cT4N1M0). The tumor was infiltrating the pelvic wall, and it was expected that establishing a safe dissection plane would be challenging with upfront surgery. Therefore, he first underwent three courses of neoadjuvant chemotherapy, which resulted in tumor shrinkage. Although no invasion of the tumor into the ureter was observed, the ureter's course was in close proximity, raising concerns about intraoperative injury. Consequently, the surgery was performed with intraoperative navigation using a fluorescent ureteral stent, which was visualized intraoperatively. The operation was successfully performed by maintaining an appropriate dissection layer, ensuring safety. The postoperative pathological examination confirmed that the surgical margins were negative, and two years have elapsed since the surgery, no recurrence has been observed. In this way, fluorescent ureteral catheters facilitate the visualization of the appropriate dissection layer, ultimately helping to prevent injury to both the urinary tract and blood vessels. They are particularly useful for ensuring safe surgery when tumors are huge or when preoperative adjuvant therapies have been administered, resulting in tissue hypertrophy and fibrosis. Under such conditions, these stents are considered to significantly contribute to the safety of the procedure.

## Introduction

Robotic-assisted surgery for colorectal cancer has become increasingly common, and it has also been utilized for advanced stages of colorectal cancer [1]. However, when the tumor is large, the risk of misidentification during dissection is high, and in robot-assisted surgery without tactile sensation, caution must be exercised to avoid damage to other organs. In recent years, navigation surgery using fluorescent ureteral catheters that emit fluorescence upon near-infrared light irradiation has been gaining attention. We report a case in which a safe surgical procedure was performed using a fluorescent ureteral catheter for advanced sigmoid colon cancer. Written informed consent has been obtained from the patient to publish this paper.

## Case presentation

The case was a 65-year-old Japanese man who presented to our hospital with abdominal pain as his chief complaint. The patient’s vital signs were as follows: body temperature 37°C, blood pressure 110/70 mmHg, and pulse rate 90 beats per minute. No anemia or jaundice was observed. There was a distension and tenderness in the lower abdomen, but rebound tenderness was not present. Blood tests showed a white blood cell count (WBC) of 8,800/μL, C-reactive protein (CRP) of 2.61 mg/dL, and an increased inflammatory response (Table 1).

**Table 1:** Laboratory data WBC: white blood cell; RBC: red blood cell; HGB: hemoglobin; HCT: hematocrit; CRP: C-reactive protein; LDH: lactate dehydrogenase; AST: aspartate aminotransferase; ALT: alanine aminotransferase; T-bil: total bilirubin; D-bil: direct bilirubin; BUN: blood urea nitrogen; Na: sodium; Ki: potassium; Cl: chloride; Lac: lactate

Test (unit)	Admission value	Reference range
WBC (x10^3^/µL)	8.8	3.5-8.0
RBC (x10^6^/µL)	4.58	3.8-4.8
HGB (g/dL)	13.9	13.1-16.3
HCT (%)	45	36.0-47.0
Platelets (x10^3^/µL)	318	120-400
CRP (mg/dL)	2.61	< 0.14
Total protein (g/dL)	7.8	6.5-8.2
Serum albumin	4.3	3.7-5.5
LDH (U/L)	192	124-222
AST (IU/L)	38	10-40
ALT (IU/L)	31	5-45
T-bil (mg/dL)	0.7	0.4-1.5
D-bil (mg/dL)	0.3	0.0-0.4
BUN (mg/dL)	9.5	8.0-20.0
Serum creatinine (mg/dL)	0.69	0.61-1.04
Na (mEq/L)	141	138-145
K (mEq/L)	4.1	3.5-5.0
Cl (mEq/L)	102	97-108
Lac (mmol/L)	0.8	0.5-1.6

**Figure 1:**
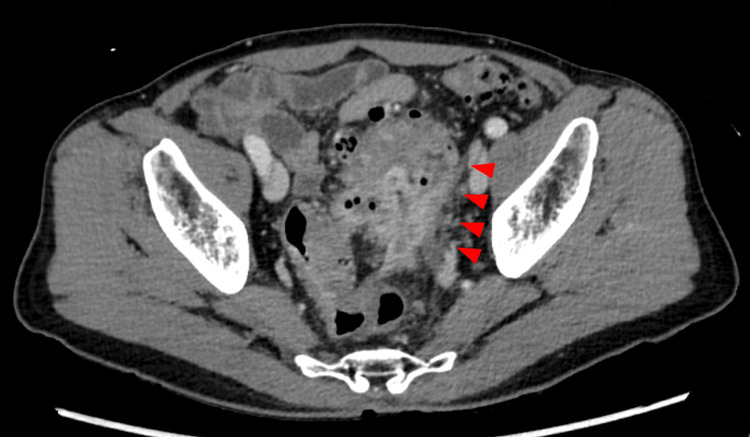
Abdominal computed tomography show that the local advanced tumor was adjacent to the left pelvic wall (red arrowheads)

CT imaging showed that the tumor was over 5 cm and was adjacent to the left pelvic wall (Figure 1).

**Figure 2:**
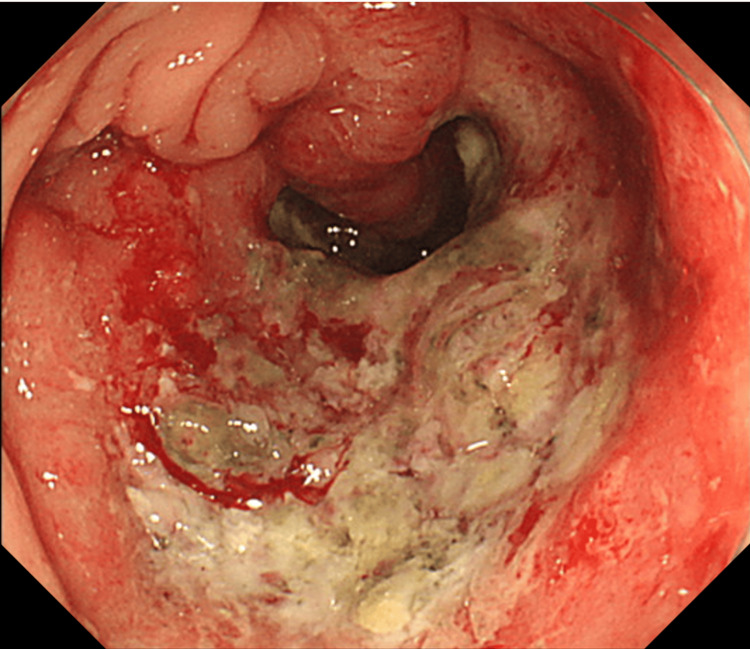
A colonoscopy revealed a type 2 colon cancer in the sigmoid colon

A colonoscopy revealed sigmoid colon cancer (Figure 2).

**Figure 3:**
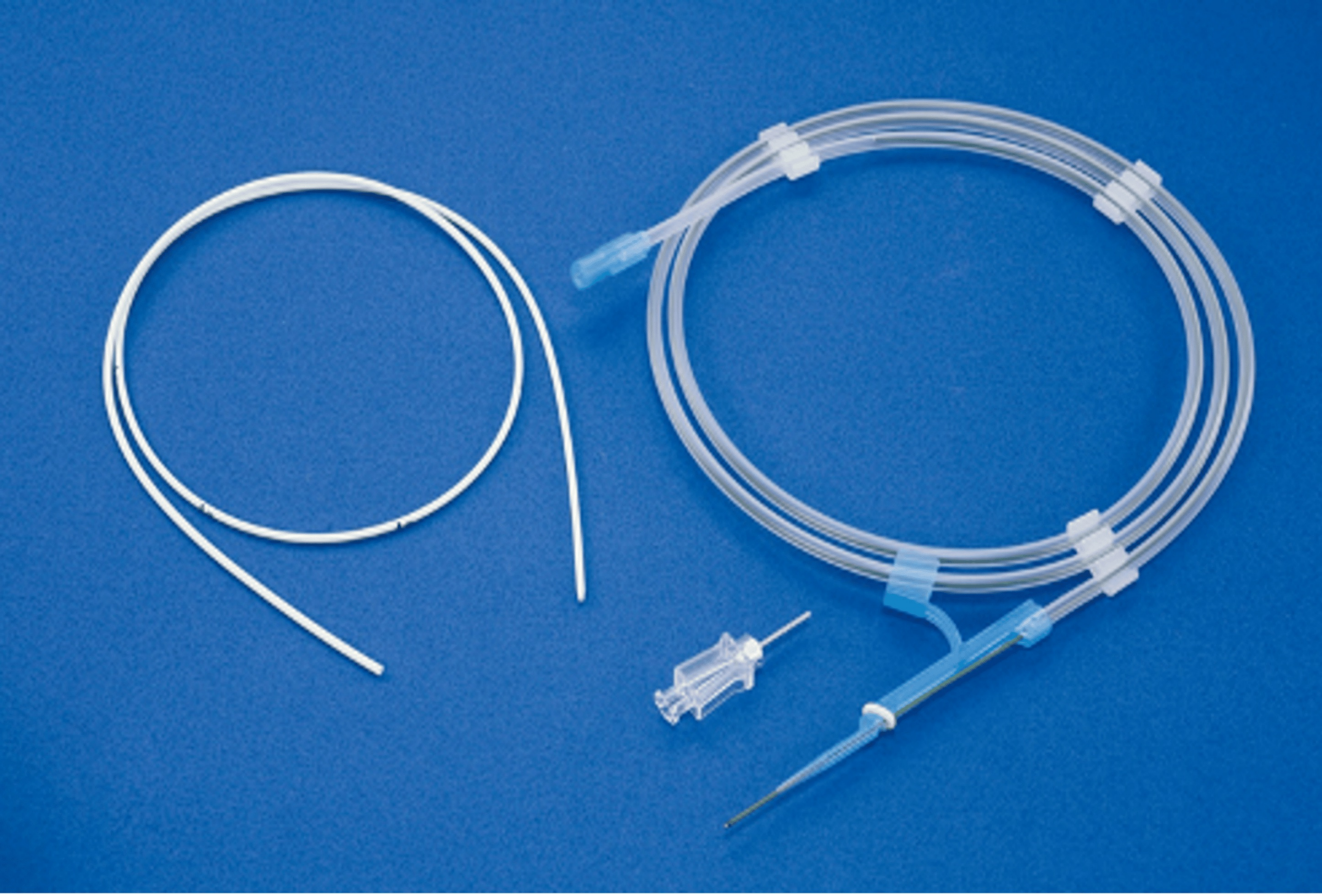
Fluorescent ureteral catheter (Near Infrared Catheter NIRC™, Cardinal Health, Inc., Tokyo, Japan)

Although left hydronephrosis was not observed, the possibility of left ureteral invasion could not be ruled out. Therefore, the patient first underwent three courses of neoadjuvant chemotherapy (three courses of CAPOX, i.e., capecitabine and oxaliplatin), which resulted in tumor shrinkage. Although a subsequent CT scan after chemotherapy revealed no evidence of invasion into the left ureter, the ureter's course was in close proximity, raising concerns about intraoperative injury. To avoid intraoperative ureteral injury, we decided to perform the surgery using a fluorescent ureteral stent. After general anesthesia, the patient was positioned in the lithotomy position, and the urology surgeon performed cystoscopy with fluoroscopic guidance. A guidewire was inserted into the left ureteropelvic junction, and a fluorescent ureteral catheter (Near Infrared Catheter NIRC™, Cardinal Health, Inc., Tokyo, Japan) was inserted and left in place (Figure 3).

**Figure 4:**
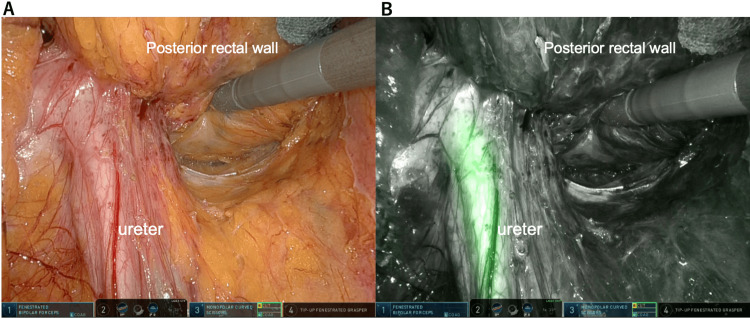
The left ureter is adjacent to the tumor (A); the course of the ureter becomes clear with the use of a fluorescent ureteral stent (B)

The procedure took 15 minutes. Next, we began the robotic surgery. The tumor was located in the sigmoid colon and was adherent and infiltrating the left pelvic wall (Figures 4A, 4B).

**Figure 5:**
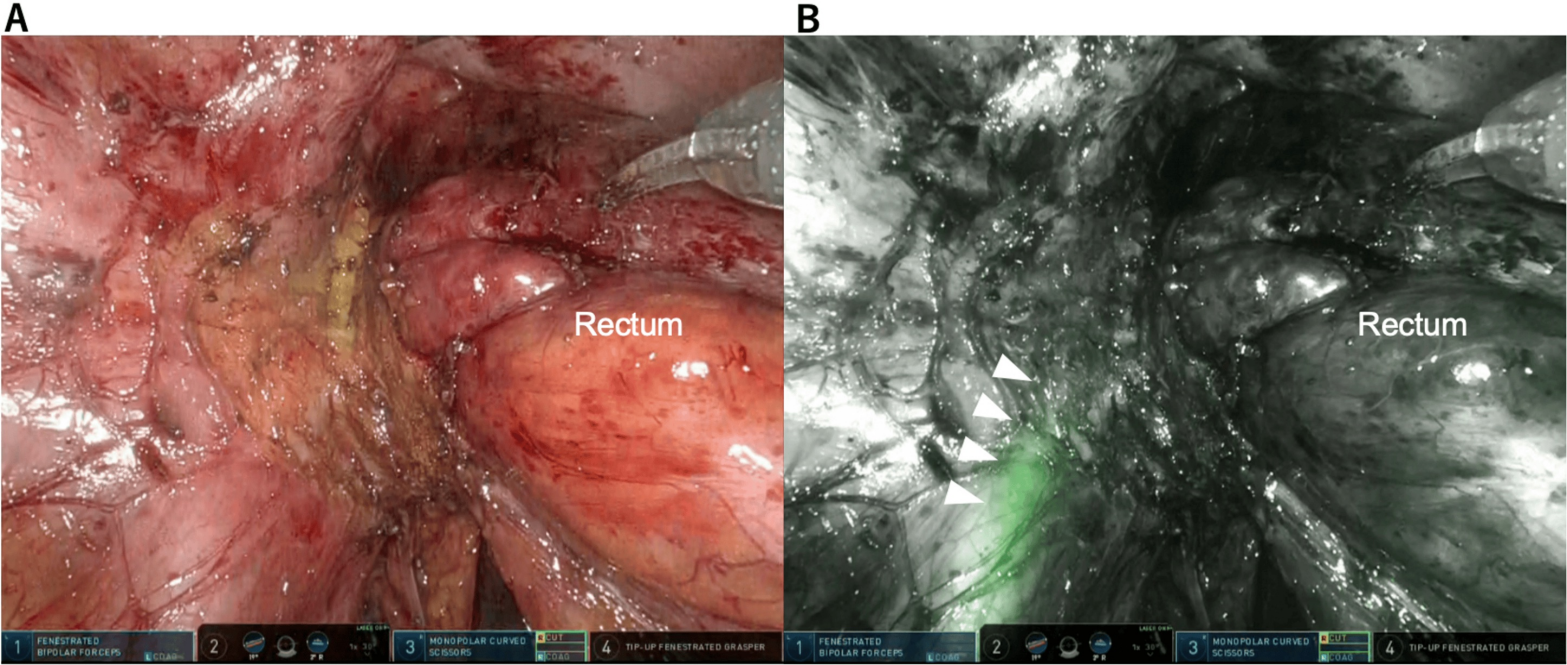
The tumor was adjacent to the ureter and the boundary between them is unclear (A), but the ureter (white arrowheads) was visible under near-infrared light (B)

The tumor was adjacent to the ureter, and the boundary between them is unclear, but the ureter was visible under near-infrared light, allowing for its safe preservation (Figures 5A, 5B).

**Figure 6:**
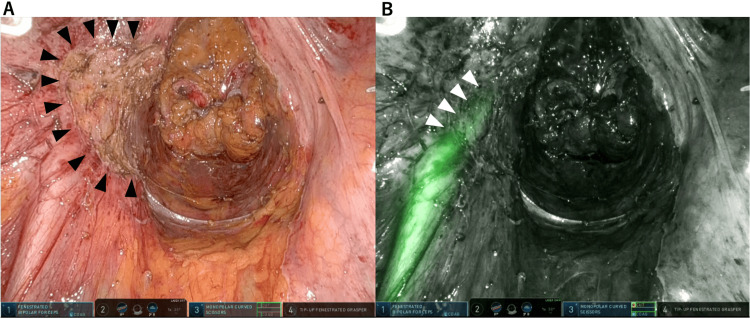
The tumor that had invaded the left pelvic wall was completely removed (A, black arrowheads); the ureter was safely preserved (B, white arrowheads)

The affected area was completely resected, and the anastomosis was performed (Figures 6A, 6B).

**Figure 7:**
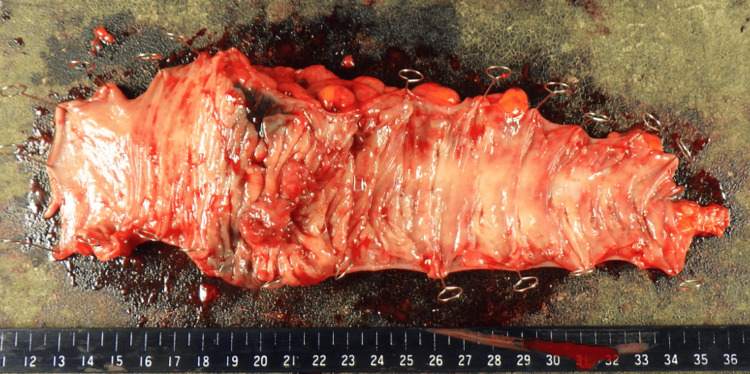
Macroscopic findings of the primary sigmoid colon cancer; the tumor, which had shrunk to approximately 2×3 cm following preoperative chemotherapy, was seen in the resected sigmoid colon and rectum

The operative time was 174 min, with intraoperative bleeding of 5 mL. Macroscopic examination of the resected specimen revealed a type 2 tumor, which had shrunk to approximately 2×3 cm following preoperative chemotherapy (Figure 7).

**Figure 8:**
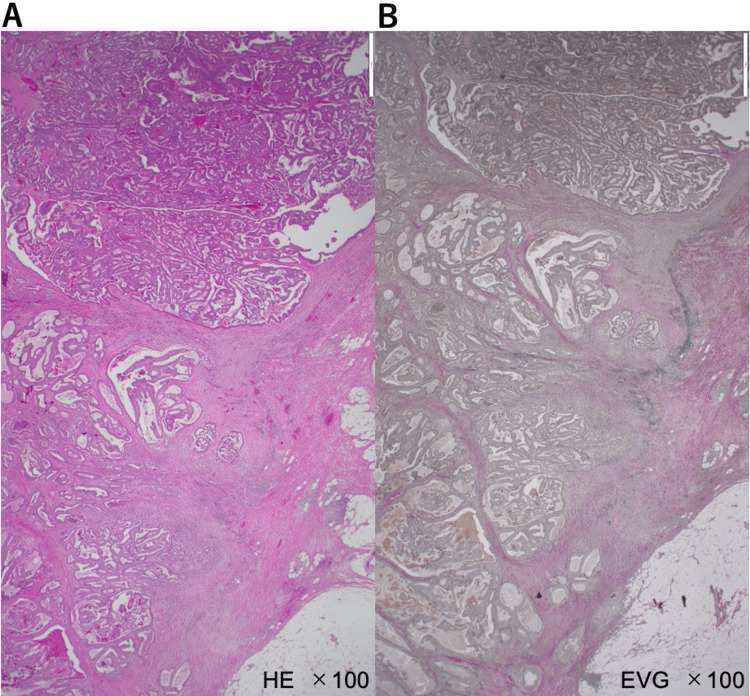
Histopathological findings of hematoxylin-eosin (HE) stain (A), and those of Elastica van Gieson (EVG) stain (B); no tumor was visible at the resection margin, confirming a negative margin

Histopathological examination revealed that the tumor, tubular adenocarcinoma, moderately differentiated type, was completely excised, and the margins were negative (Figures 8A, 8B).

According to the 8th edition of the TNM classification, the pathological stage of the cancer was T4aN1aM0 stage IIIB [2]. The postoperative course was uneventful, and the patient resumed eating on postoperative day (POD) 3 and was discharged on POD 7. He underwent three additional courses of CAPOX one month after surgery. At present, two years postoperatively, no recurrence has been observed.

## Discussion

The incidence of iatrogenic ureteral injury during colorectal surgery has been reported to be 0.3-1.5% [3,4]. Conventional ureteral catheter stents are useful for preventing ureteral injury during open surgery, but their effectiveness is limited in robotic surgery, where tactile feedback is absent and visualizing the ureteral catheter is difficult [5,6]. The primary causes of ureteral injury during surgery are electrocoagulation and ultrasonic coagulation cutting devices. The most common sites for ureteral injury are reported to be the region where the common iliac artery enters the pelvic cavity, the crossing point with the uterine artery, and the vicinity of the bladder. Even minor ureteral injuries can lead to complications such as necrosis, obstruction, or stricture. Therefore, when ureteral injury is identified during surgery, ureteral anastomosis or ureterovesical reimplantation is often performed depending on the extent of the injury [5]. This requires extending the surgical time and placing a ureteral stent. Furthermore, periodic replacement of the ureteral stent may be required postoperatively [7].

In recent years, NIRC™ (Cardinal Health, Inc., Tokyo, Japan) containing Indocyanine green (ICG) components has been developed for ureteral catheters, and it has been reported that the ureter can be visualized by observing it with near-infrared light [8,9]. Cases of fluorescent ureteral catheter use in colorectal surgery have been reported sporadically for colon cancer and diverticulitis [9,10]. While conventional ureteral catheters allowed for the identification of the ureter's position through palpation during surgery, palpation is difficult in robotic surgeries. In contrast, fluorescent ureteral catheters can be visualized up to a depth of approximately 5 to 10 mm, making them effective even in robotic surgeries where palpation is not possible [9]. A previous study reported that the ureter could be identified in 91.3% of cases using fluorescent ureteral catheters during colorectal surgery [11]. In addition, the identification of the fluorescent ureteral catheters that use near-infrared light is simple and easy with only the firefly mode. Furthermore, they enable intraoperative assessment of ureteral injury, which may contribute to the early detection and prevention of ureteral damage postoperatively. A fluorescent ureteral catheter may improve the outcome of ureteral injuries.

On the other hand, there are the following issues. Firstly, the near-infrared light penetration depth in biological tissue is reported to be 5-10 mm, and depending on the tissue thickness, the ureter may not be visible. Visualization of the ureter as it traverses deep layers such as the ureterovesical junction is unclear. Therefore, when the tumor is located near the bladder, the appropriateness of a fluorescent ureteral catheter placement must be carefully assessed. Secondly, in Japan, fluorescent ureteral stents are not covered by the universal health insurance system and are very expensive. No reports mention the cost-effectiveness of fluorescent ureteral catheters, but there are reports indicating that the use of ureteral catheters for colon resection surgery significantly prolonged hospital stays and increased medical costs [12]. We remove a fluorescent ureteral catheter used during surgery the day after colorectal surgery, taking measures to ensure that catheter placement does not prolong the hospital stay. From a medical cost perspective, the indications for placing a fluorescent ureteral stent should be limited. We carefully evaluate the indications and use it selectively. Thirdly, there is the issue of extended patient time in the operating room. Since X-ray fluoroscopy and cystoscopy equipment are required for ureteral catheter insertion, preparation time is necessary. However, in reality, the additional time required to insert the fluorescent ureteral catheter was 15 minutes in this case, which is considered fully acceptable. Considering the aforementioned issues, it is crucial to thoroughly evaluate whether stenting is appropriate before proceeding with stent placement.

This study has a few limitations. The patient has only been followed for two years postoperatively, making the long-term prognosis insufficiently evaluated. Another limitation is that we have not been able to determine whether there is a difference in surgical outcomes between cases where a fluorescent ureteral catheter was used and those where it was not.

Navigation surgery using near-infrared light has been reported to potentially avoid iatrogenic damage to other organs and reduce stress for surgeons during procedures. Fluorescent ureteral stents were considered useful as a device for surgery near the ureter.

## Conclusions

We experienced a case where a robot-assisted sigmoid colon resection was safely performed using a fluorescent ureteral catheter. The fluorescent ureteral catheter enables visualization of the ureter during surgery and is considered useful for avoiding ureteral injury. Further consideration is needed regarding its indications, including safety and cost aspects.

Considering the aging society, locally advanced colorectal cancer is likely to increase further in the future. It is necessary to effectively integrate minimally invasive procedures such as robotic surgery with navigation surgery to ensure curative potential and safety while maintaining patient quality of life postoperatively.
